# Left Atrium Reverse Remodeling in Fusion CRT Pacing: Implications in Cardiac Resynchronization Response and Atrial Fibrillation Incidence

**DOI:** 10.3390/jcm13164814

**Published:** 2024-08-15

**Authors:** Cristina Văcărescu, Dragoș Cozma, Simina Crișan, Dan Gaiță, Debora-Delia Anutoni, Mădălin-Marius Margan, Adelina-Andreea Faur-Grigori, Romina Roteliuc, Silvia-Ana Luca, Mihai-Andrei Lazăr, Oana Pătru, Liviu Cirin, Petru Baneu, Constantin-Tudor Luca

**Affiliations:** 1 Department of Cardiology, “Victor Babeș” University of Medicine and Pharmacy, 300041 Timisoara, Romania; cristina.vacarescu@umft.ro (C.V.); simina.crisan@umft.ro (S.C.); dan.gaita@umft.ro (D.G.); silvia.luca@umft.ro (S.-A.L.); lazar.mihai@umft.ro (M.-A.L.); petru.baneu@umft.ro (P.B.); constantin.luca@umft.ro (C.-T.L.); 2 Institute of Cardiovascular Diseases Timisoara, 300310 Timisoara, Romania; anutoni.debora@cardiologie.ro (D.-D.A.); faur.andreea@cardiologie.ro (A.-A.F.-G.); roteliuc.romina@cardiologie.ro (R.R.); 3 Research Center of the Institute of Cardiovascular Diseases Timisoara, 300310 Timisoara, Romania; 4 Department of Functional Sciences, Discipline of Public Health, “Victor Babeș” University of Medicine and Pharmacy, 300041 Timisoara, Romania; margan.madalin@umft.ro; 5 Doctoral School, “Victor Babeș” University of Medicine and Pharmacy, 300041 Timisoara, Romania; oana.patru@umft.ro (O.P.); liviu.cirin@umft.ro (L.C.)

**Keywords:** cardiac resynchronization therapy, fusion pacing, left atrium remodeling, atrial fibrillation

## Abstract

**Background**: When compared to biventricular pacing, fusion CRT pacing was linked to a decreased incidence of atrial fibrillation (AF). There is a gap in the knowledge regarding exclusive fusion CRT without interference with RV pacing, and all the current data are based on populations of patients with intermittent fusion pacing. **Purpose**: To assess left atrium remodeling and AF incidence in a real-life population of permanent fusion CRT-P. **Methods**: Retrospective data were analyzed from a cohort of patients with exclusive fusion CRT-P. Device interrogation, exercise testing, transthoracic echocardiography (TE), and customized medication optimization were all part of the six-monthly individual follow-up. **Results**: Study population: 73 patients (38 males) with non-ischemic dilated cardiomyopathy aged 63.7 ± 9.3 y.o. Baseline characteristic: QRS 159.8 ± 18.2 ms; EF 27.9 ± 5.1%; mitral regurgitation was severe in 38% of patients, moderate in 47% of patients, and mild in 15% of patients; 43% had type III diastolic dysfunction (DD), 49% had type II DD, 8% had type I DD. Average follow-up was 6.4 years ± 27 months: 93% of patients were responders (including 31% super-responders); EF increased to 40.4 ± 8.5%; mitral regurgitation decreased in 69% of patients; diastolic profile improved in 64% of patients. Paroxysmal and persistent AF incidence was 11%, with only 2% of patients developing permanent AF. Regarding LA volume, statistically significant LA reverse remodeling was observed. **Conclusions**: Exclusive fusion CRT-P was associated with important LA reverse remodeling and a low incidence of AF.

## 1. Introduction

The left atrium (LA) was proposed as a marker of chronic, cumulative effects of underlying left ventricle (LV) dysfunction in the same way that glycated hemoglobin shows diabetes status [1]. Moreover, a reduction in LA function has been identified in all stages of HF as an independent predictor of adverse events, including death [2].

LA volume has been shown to be a robust and accurate predictor of cardiovascular outcomes [3], but the relation between size and LA function in cardiac resynchronization therapy (CRT) patients is still not fully understood. CRT and LA functions and dimensions have been studied as a bidirectional connection, and LA function appears to be a good predictor of CRT response [4]. CRT induces LA anatomic, electrical, and structural reverse remodeling [5,6]. However, these data are validated in biventricular CRT pacing (BiV pacing).

When compared to biventricular (BiV) pacing, the adaptive CRT algorithm favors LV fusion pacing, or LV-only pacing, at a heart rate <100 b/min, and has been linked to a lower incidence of atrial fibrillation and important LA reverse remodeling [7]. Fusion CRT pacing (CRT-P), using dual chamber devices without an RV lead, represents an elegant and safe alternative in selected patients [8], with the obvious condition of maintaining sinus rhythm. Moreover, fusion CRT-P was associated with a high number of responders and super-responders [9], and was proposed as an alternative to increase the CRT response in non-responder patients with BiV pacing [10].

As far as we currently know, there is no information available on reverse remodeling of the left atrium in LV-only pacing CRT without a right ventricular (RV) lead. The purpose of this study was to assess LA remodeling regarding size and function in a real-life situation of permanent fusion pacing CRT in patients with LV-only pacing without an RV lead. The hypothesis of an important left atrium remodeling associated with LV fusion pacing (without possible interference of RV pacing) could be a main determinant in reducing atrial fibrillation (AF) incidence in the CRT population. 

## 2. Material and Methods

### 2.1. Study Design and Population

Retrospective data were analyzed from a cohort of patients with non-ischemic dilated cardiomyopathy and CRT-P indication, implanted with right atrium/left ventricle (RA/LV)-only DDD pacing systems. Classical indications, according to current guidelines, were used to assess patients for CRT-P implantation. The following criteria were used to perform exclusive fusion CRT pacing: sinus rhythm with preserved AV conduction (PR interval < 240 ms), heart failure NYHA class II and III, EF < 35%, and LBBB QRS morphology with a QRS duration > 130 ms. The following were considered exclusion criteria for implanting a RA/LV DDD pacing system: ischemic etiology of cardiomyopathy, HF class NYHA I or IV, history of persistent or paroxysmal atrial fibrillation, history of syncope, the presence of conduction system disease (type I AV block with PR > 240 ms, type II and III AV block), or the presence of structural cardiomyopathies or channelopathies associated with risk of sudden cardiac death that would require a defibrillator lead.

Every six months, there was a complete follow-up (FU) that included transthoracic echocardiography, an exercise test, device interrogation, and customized medication optimization. Prior to implantation, all patients received optimal HF medical therapy, which included ACE inhibitor/ARB, SGLT2 inhibitors, antialdosteronics, fixed combination sacubitril/valsartan, and betablockers/ivabradine. Each patient’s dosage was adjusted based on clinical and paraclinical variables (heart rate, blood pressure, renal function). Regarding the CRT response, patients were divided into 3 groups: responders (including super-responders), hypo-responders and non-responders. Responder patients were defined as those with an increase in LVEF > 5% and a decrease in LV end systolic volume ≥15%, along with a clinical response (improvement in NYHA functional class) [11]. Super-responders were defined as patients with a stable ejection LVEF ≥ 45% and no signs of HF after fusion CRT-P. Hypo-responders were defined as patients with no significant echocardiographic response, but with a favorable clinical CRT response. In non-responder patients, no clinical or echocardiographical improvement was noted. 

All clinical cardiological and biological events were recorded during follow-up: hospitalization for decompensated HF, ventricular and supraventricular tachyarrhythmias, changes in medication, changes in device programming, and significant biological impairment. Medication optimization and device programming were conducted according to an internal protocol adapted for fusion CRT pacing patients [12].

Atrial fibrillation was defined according to guideline definitions as paroxysmal, persistent, and permanent. Atrial high-rate episodes (AHREs) diagnosed on device interrogation were taken into account as paroxysmal AF when they lasted at least 5 min. 

The Helsinki Declaration of 1964 was followed throughout the study, and the Timisoara Institute of Cardiovascular Diseases’ Ethical Review Committee approved the protocol (1622/26 March 2014). Every patient involved in the study signed a written informed consent form.

### 2.2. Echocardiography Measurements

A VIVID 9 system (GE Health Medical, Milwaukee, WI, USA, 2.5 MHz transducer) was used to perform transthoracic echocardiographic evaluation. Standard views and techniques were used, and each patient’s ECG was simultaneously recorded. 

Classical measurements were performed for all patients: interventricular septum (IVS), LV end-diastolic and end-systolic diameters (LVEDD, LVESD), LV volumes (LVEDV, LVESV), and LV ejection fraction (LVEF, determined based on Simpson’s modified rule). Diastolic function was assessed by transmitral flow parameters—maximum velocities of E and A waves, and E/A ratio. Diastolic dysfunction (DD) severity was graded as mild for E/A ≤ 0.8 (type I DD), moderate (type II DD) for E/A ≥ 0.8, or severe (type III DD) if E/A ≥ 2.

Mitral regurgitation and all other valvulopathies were classified as mild, moderate, or severe according to the recommendations of the European Association of Cardiovascular Imaging [13]. Maximal tricuspid regurgitation velocity, inferior vena cava diameter, and inspiratory collapse were used to assess systolic pulmonary artery pressure (SPAS). 

Atrioventricular, interventricular, and intraventricular synchrony parameters were also assessed in all patients. The atrioventricular (AV) asynchronism was defined as inadequate timing between the end of atrial systole and the beginning of ventricular systole. We calculated the AV asynchronism as the ratio between the duration of LV filling (using pulsed Doppler at the level of the transmitral flow) and the duration of the cardiac cycle, with a cut-off value of <40% for AV asynchronism. The interventricular asynchronism was calculated as the difference between aortic pre-ejection time and pulmonary artery pre-ejection time, with a cut-off value of >40 ms for positive interventricular asynchronism. The intraventricular asynchronism represents a contraction heterogeneity between different LV segments, and many qualitative and quantitative parameters can be used to assess this type of asynchronism. However, due to the limitations regarding an optimal ultrasound window and time efficiency, we have mainly used septal to posterior wall motion delay (SPWMD) and septal-to-lateral delay (SLD) using Tissue Doppler Imaging. The cut-off value for positive intraventricular asynchronism was >130 ms for SPWMD and >60 ms for SLD [14].

All LA measurements were performed at end-systole, just before mitral valve opening (maximal size). LA volume (LAV) was assessed in the apical 4-chamber view by tracing the endocardial border cavity. The optimal 4-chamber view required for LA measurements was considered when visualization of the maximal number of pulmonary veins was possible [15]. Every attempt was made to guarantee the largest possible LA size evaluation. In situations where the shape was ambiguous, two impartial observers visually assessed the situation before a decision was made by consensus. Left atrium volume index (LAVI) was calculated in all patients using body surface area.

### 2.3. Statistical Analysis

Statistical analysis was performed using R version 4.2.0 (R Foundation for Statistical Computing, Vienna, Austria). The R Base Package was used for data manipulation, and the R Stats Package was used for performing linear regression between numerical variables. The R “ggplot2” Package (version 3.5.1) was used for plotting and graph generation. Mean ± standard deviation is used to express continuous values, while proportions were utilized to express categorical variables. The unpaired t test (for regularly distributed variables) or the Mann–Whitney U test (for non-normally distributed variables) was used to compare continuous variables between groups. To compare proportions, the chi-square test and Fischer’s exact test were employed. A *p* value < 0.05 was considered statistically significant. For linear regression specifically, we used R version 3.6.1 with the “lm” function. The dependent variables were plotted on the *y*-axis, while the independent variables (predictors) were plotted on the *x*-axis. We checked for assumptions of linearity, independence (using the Durbin–Watson statistic), homoscedasticity (examining the residual plot), and normality (Shapiro–Wilk test). Multicollinearity was evaluated using the variance inflation factor (VIF). Model fit was assessed using R-squared, adjusted R-squared, and the significance of the regression coefficients. 

## 3. Results

The study group included 73 patients implanted with RA/LV DDD pacing systems at Timisoara Institute of Cardiovascular Diseases between 2012 and 2022. Non-ischemic dilated cardiomyopathy and LBBB morphology with QRS complex >130 ms was documented in all patients. Baseline demographic data and medical therapy are presented in Table 1.

All devices were programmed at a rest rate of 60 beats/min with an individualized AV interval programming (AV paced 145 ± 23 ms, AV sensed 115 ± 26 ms) that allowed fusion pacing in all patients. The LV lead was placed posteriorly in 9 patients (12%), postero-laterally in 28 patients (38%), laterally in 26 patients (36%), and anterolaterally in 7 patients (10%); epicardial leads were needed in 3 patients (4%). Venography was conducted in order to determine the best site for LV lead placement. Omnipaque (iohexol 350 mg/1 mL, GE Healthcare, Chicago, IL, USA) was used as a contrast agent, with an average use of 30–50 mL/patient. Contrast-induced nephropathy, defined as an increase in serum creatinine ≥0.3 mg/dL (at 24/48 h after implantation), was documented in six patients (8%), all of them with previous chronic kidney disease. None of these patients needed intensive nephrological interventions; in all the cases, the serum creatinine was reduced and stabilized using proper i.v. hydroelectrolytic rebalancing and a temporary stop of possibly nephrotoxic medication. 

During follow-up, more than 400 exercise tests were performed in all patients, with a mean of 123 ± 19 Watts (6.7 ± 1.4 METS). Following the exercise tests, 38% of patients needed CRT device reprogramming (changes in sensed/paced AV delay or maximum tracking rate), while in 36% of patients, medication optimization was performed (special focus on beta blockers and ivabradine) to ensure constant fusion pacing. 

At the baseline evaluation, the mean ejection fraction (EF) was 27.9 ± 5.1%. Mitral regurgitation was severe in 28 patients (38%), moderate in 34 patients (47%), and mild in 11 patients (15%). Regarding LA function, 31 patients (43%) had type III diastolic dysfunction, 36 patients (49%) had type II DD, and 6 patients (8%) had type I DD. Detailed baseline echocardiography data are presented in Table 2.

The average follow-up (FU) was 6.4 years ± 27 months, with the longest FU period of 14 years and the shortest FU of 2 years. Regarding the CRT response, as defined in the Methods Section, 93% of patients were responders (including 31% super-responders), 4% were hypo-responders, and 3% of patients were non-responders. The mean FU time to achieve the CRT response was between 3 and 6 months after fusion CRT-P. LVEF increased at 40.4 ± 8.5% and NYHA functional class improved with at least one grade in all responder patients. Mitral regurgitation decreased in 51 patients (69%) and diastolic profile improved in 47 patients (64%). Detailed data regarding the statistical analysis between echocardiographycal parameters at baseline and during the FU are presented in Table 3. 

**Figure 1:**
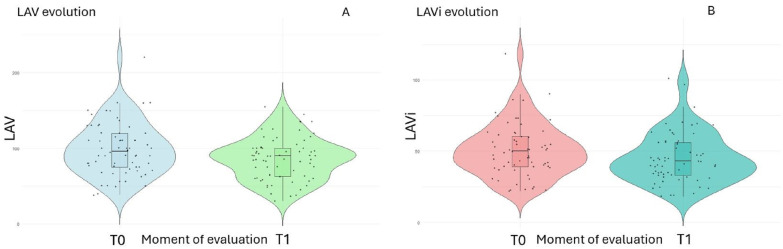
Violin plots showing LAV evolution (**A**) and LAVi evolution (**B**) at T0 (the moment of fusion CRT-P implantation) and T1 (average follow-up of 6.4 years ± 27 months).

**Figure 2:**
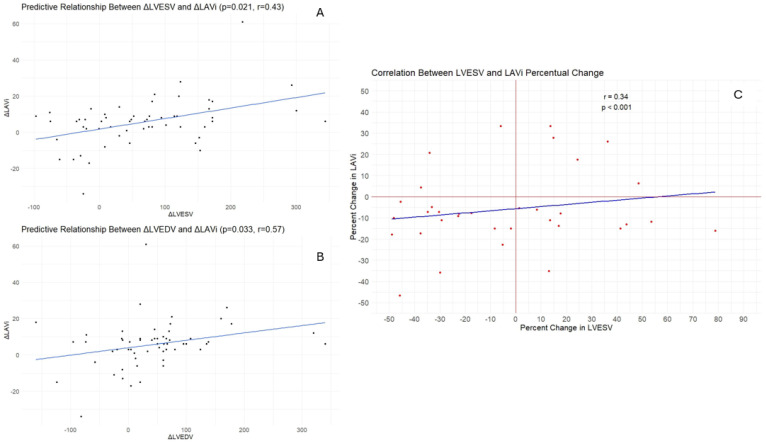
Linear regression analysis showing significant statistical correlations between changes in absolute values of LVESV/LVEDV and LAVi (**A**,**B**) and percent change between LVESV and LAVi (**C**). The dependent variables were Δ LAVi and LAVi percent change, while the independent variables were Δ LVESV, Δ LVEDV and percent change in LVESV. Δ = delta.

The evolution of LAV and LAVi are presented in Figure 1A,B. Linear regression analysis was used to evaluate the relationship between changes in LVEDV/LVESV and changes in LAVi (Figure 2A,B). Furthermore, a significant statistical correlation was noted regarding the percentage change in LAVi versus LVESV (Figure 2C).

The overall incidence of atrial fibrillation was 13%, with an incidence of 4% paroxysmal AF (including AHRE), 7% persistent AF, and 2% permanent AF. Patients with permanent AF were upgraded to a biventricular device and a rate control strategy was chosen. For patients with paroxysmal and persistent AF, different classes of antiarrhythmics were used, and electroconversion when needed. One patient (1%) with paroxysmal AF underwent ablation with pulmonary vein isolation, and another two patients (3%) developed typical atrial flutter and cavotricuspid isthmus ablation was performed. The outcome was positive for all these patients, without any other complications.

No ventricular tachyarrhythmias were noted during FU and there was no need for an upgrade to a CRT-D device in our group of patients. Three patients (4%) on medical treatment with beta blockers and amiodarone for persistent atrial fibrillation developed complete AV block and needed an upgrade to a triple chamber CRT-P device. Six patients (8%) had a pacemaker replacement due to an elective replacement indicator.

Five patients (7%) died during FU: two patients due to decompensated, refractory HF (both of them were non-responders to CRT with severe LA enlargement and type III DD); two patients due to complicated bronchopneumonia and septic shock; and one patient due to end-stage leukemia. No deaths were recorded in the super-responders group. Worsening heart failure was noted in 17 patients (23%) who needed readmission to the hospital; the main causes of HF decompensation were persistent AF and atrial flutter, respiratory infections, medication, and diet issues.

## 4. Discussions

Our study showed an important process of left atrium remodeling and a low incidence of atrial fibrillation in patients with fusion CRT pacing. To the best of our knowledge, this is the first study that analyzed the incidence of atrial fibrillation in a population with exclusive fusion CRT-P without any interference with RV pacing. Interestingly, these results correlate with a high number of responder and super-responder patients. 

There are several studies that have proved that a higher RV pacing burden adversely affects LA structure and impairs its function, leading to an increased risk of AF development [16,17,18,19]. Conventional RV pacing causes LV dyssynchrony due to RV preexcitation and reduced LV compliance followed by an increased filling pressure, resulting in LA remodeling, which is the substrate for AF [20,21]. Xie et al. observed that chronic RV pacing results in a reduction in the total and passive emptying fraction of LA, and therefore a reduced atrial pump function and a larger volume of the atrium [17]. 

An important promoter of fusion pacing, the adaptive CRT algorithm was associated with improved patient survival and lower incidence of AF in a real-world, prospective, nonrandomized registry [22]. A post hoc analysis from an adaptive CRT trial demonstrated that conventional CRT with non-physiological pacing AV intervals (too short or too long) is associated with higher risk of long-duration AF compared with fusion CRT, suggesting that the continuous optimization of AV pacing intervals results in AV synchrony, with a maximum benefit for patients with long PR intervals [8]. On the same note, a prospective randomized study that used the same algorithm obtained similar results, but the super-responders’ rate was higher in the LV-only pacing group (68.4%) compared with the BiV pacing group (36.4%) [23].

Research on the use of LV fusion CRT pacing in non-RV lead settings is lacking. Although non-inferior to BIV pacing [24,25], LV-only pacing is not commonly employed in clinical practice. The biggest concern regarding fusion pacing CRT is related to atrioventricular variability of conduction and inconstant pacing with lack of ventricular capture. Nevertheless, the presence of an RV lead does not guarantee adequate LV pacing and capture. In both clinical practice, and also as a future area of research, a difference has to be made between biventricular pacing (with or without AV and VV interval optimization), intermittent fusion pacing (as provided by algorithms like adaptive CRT from Medtronic [7] or SmartDelay from Boston Scientific [26] in triple chamber devices), and exclusive fusion pacing. One of the strengths of our study is related to the fact that we have provided exclusive fusion pacing for a relative long period of time with a favorable outcome. 

In the era of conduction pacing system, the classical CRT with biventricular pacing provides a “non-physiological resynchronization” due to prolonged electrical activation and mechanical asynchronism associated with RV pacing. Despite developments in this field, response rates and the durability of therapy remain essentially stable, still with a 30% rate of non-responders. Suboptimal atrioventricular timing could be the most common modifiable factor impacting clinical outcomes of CRT. The degree of the QRS duration reduction with CRT correlates with better results, and the combination of LV fusion pacing with intrinsic conduction activation can result in greater QRS narrowing [27]. 

Even if the newer techniques such as His bundle pacing and LBBB pacing have gained more and more ground in the field of physiological pacing, there are still challenges and troubleshooting to overcome [28]. At the present time, all these techniques are rather complementary to classical biventricular CRT. The 2013 ESC cardiac pacing and resynchronization guideline stated that LV-only fusion CRT pacing is non-inferior to BiV pacing [29]. The 2021 ESC guideline lacks guidance on fusion pacing, indicating a significant knowledge gap in this field [30]. Nevertheless, the area of fusion pacing is still of interest in CRT, and recent research is showing favorable outcomes and even superiority of fusion pacing compared to biventricular pacing [23,27,31].

The SAVE PACe trial showed that patients with sinus-node disease who received dual-chamber minimal RV pacing had a reduced risk of persistent AF compared with those with conventional dual-chamber pacing. A lower RV pacing rate prevents ventricular desynchronization, through AV synchrony preservation. In this large trial, minimal ventricular pacing caused a 40% reduction in relative risk and a 4.8% reduction in absolute risk, when referring to AF development [32]. Another study by Wang et al. [18] indicated a change in LA diameter—a reduction for those patients with left bundle branch area pacing, and on the contrary, an increase for those with RV pacing. Moreover, RV pacing led to a negative change in LVEF. On a similar note, in a more recent study, Ravi et al. demonstrated that a higher burden of RV pacing is more likely associated with new-onset AF, due to reduced LV compliance and dyssynchrony [20].

Pastore et al. evaluated retrospectively the occurrence of AF during different pacing sites—Hisian area, RV septal, and RV apical—in 477 patients with advanced or complete AV block. AF occurrence was noticed in 114 patients (23.9%), the lower incidence being observed in the Hisian area pacing group (16.9%), compared with RV septal (25.7%) or RV apical (28.0%) groups. A more physiological LV activation, which is obtained during Hisian area pacing, has an important role in LA function preservation, decreasing the risk of LA remodeling and, therefore, AF occurrence [16]. The long Min-VPACE study showed that minimal RVP reduced AF burden—12.8 ±1 5.3% in minimal RVP, compared with 46.6 ± 42.2% in the cohort with high burden RVP, and consequently the risk of progression from paroxysmal to persistent AF [33].

A matter of concern regarding RA/LV-only pacing devices is the risk of advanced/total AV block progression, which was previously reported in a large population study as 0.8% per year for patients with left bundle branch block and 0.5% for those without this condition [34]. Therefore, in this case, an RV lead implantation will be requested. Indeed, in our study, three patients (4%) developed AV block following antiarrhythmic and high dose beta blocker treatment, and they were upgraded to a triple chamber device. Nevertheless, the simple presence of an RV lead does not guarantee a better outcome regarding the CRT response. 

Another indication for an RV lead would be the need for a defibrillator. Indeed, the real-life choice between a CRT-P, a CRT-D, or even palliative care for patients with advanced heart failure can be difficult, with multiple challenges for the patient, the family, and the caregivers [35]. However, the indication for an ICD in primary prevention in non-ischemic DCM has decreased following the Danish trial era [36]. Our data support the results from the Danish trial, since in our group of patients with non-ischemic DCM, there was no need for a CRT-D upgrade. 

A smaller study that evaluated acute changes in LA size and function immediately after CRT showed that LAVI may be reduced within days after the implant procedure in responders to CRT [37]. A prolonged interatrial conduction time directly evaluated by electrophysiology study, as a well-known factor in the pathophysiology of atrial fibrillation, was correlated with simple Doppler measurements [38], so we can assume that morphological reverse remodeling of the LA is associated with electrical reverse remodeling, and thus a lower incidence of AF. Emerging evidence was published recently in a meta-analysis that evaluated a total of 2191 patients recruited in 10 studies with mean follow-up duration of 10.5 months; the conclusion was that baseline LAVI predicts CRT response, and its reduction reflects LA remodeling [39]. Additionally, more and more data support the relationship between CRT and LA phasic function, evaluated specifically by LA strain [21]. An increase in LA reservoir strain was associated with favorable long-term outcome after CRT [40,41]. 

Additionally, we have to take into account the possible influence of contrast-induced nephropathy after implantation as a possible determinant of the CRT response [42]. This can be attributed to the close and bidirectional connection between renal and cardiac functions: a decrease in cardiac output can impair renal function, while kidney damage can also lead to a decline in cardiac performance. However, the incidence of contrast-induced nephropathy in our study group was relatively low, without the need for major nephrological interventions.

As a future perspective, our study, along with the already existing evidence, highlights the need for a prospective, not only observational, but also randomized, trial with active interventions in device programming (fusion pacing vs. BiV pacing, no optimization vs. AV and VV interval optimization) correlated with routine evaluation of LA function and size to maximize CRT response. 

### Study Limitations

One of the main limitations is related to the lack of a control group with biventricular pacing. Due to the fact that this study was retrospective and observational, we can only present data regarding LA remodeling in fusion pacing exclusively. Nevertheless, the incidence of atrial fibrillation was lower in our group of patients compared with the existing data about biventricular CRT, as discussed above.

A possible bias regarding LA remodeling and the low incidence of atrial fibrillation is related to the high number of responders and super-responders in our group of patients. Even so, in our opinion, this reflects the bidirectional relation between LA and LV remodeling and is another argument in favor of fusion pacing CRT, along with all the other interventions performed to maximize the outcome: adequate selection of patients for CRT and periodic follow-ups, including clinical and echocardiographic evaluation, exercise tests, and device interrogation. All these led to continuous medication and device optimization, with possible implications for increasing the number of responders and super-responders. 

Another limitation is related to the relatively low number of patients included. This can be explained by the strict inclusion and exclusion criteria in order to perform exclusive fusion pacing CRT and by the innovative technique of using DDD devices. Despite the relatively small group, the long-term follow-up suggests that we had obtained reliable results in time for the patients included. Nevertheless, we agree that other parameters regarding LA evaluation, such as LA strain, would be interesting to analyze; however, in our cohort of patients, we did not have consistent available retrospective data.

## 5. Conclusions

Exclusive fusion CRT pacing was associated with important LA reverse remodeling and a low incidence of AF. Larger randomized studies are needed to validate these results and assess the role of fusion pacing in LA remodeling and AF incidence compared with biventricular pacing.

## Figures and Tables

**Table 1:** Baseline demographic data and medical therapy.

		All Patients (n = 73)
**Mean age (years), mean ± SD**		63.7 ± 9.3
Male, n (%)		38 (52%)
NYHA functional class (n, %)	II	33 (45%)
	III	40 (55%)
Electrocardiogram	PR interval (ms), mean ± SD	181.3 ± 21.9
	QRS complex (ms), mean ± SD	159.8 ± 18.2
Hypertension, n (%)		39 (53%)
Diabetes mellitus, n (%)		30 (41%)
Chronic kidney disease, n (%) *		26 (37%)
Baseline therapy	Beta blockers, n (%)	64 (88%)
	Ivabradine, n (%)	39 (53%)
	ACEI/ARB, n (%)	51 (70%)
	Diuretics, n (%)	69 (95%)
	Antialdosteronics, n (%)	62 (85%)
	Sacubitril, n (%)	20 (27%)
	SGLT2 inhibitors, n (%)	14 (19%)

* A decrease in creatinine clearance of less than 90 mL per/min was considered chronic kidney disease. In our group, no patient had a creatinine clearance lower than 30 mL/min. ACEI = angiotensin converting enzyme inhibitor; ARB = angiotensin receptor blockers, n = number, SD = standard deviation, SGLT2 = sodium-glucose 2 cotransporter 2.

**Table 2:** Baseline echocardiographic parameters.

Basic Echocardiographic Parameters	All Patients (n = 73)		All Patients (n= 73)
	Mean ± SD	Range	Asynchronism Parameters
IVS (cm)	1.1 ± 0.2	0.8–1.8	Septal flash (n, %)
LVEDD (cm)	6.4 ± 0.9	4.7–8.9	68 (93%)
LVEDV (mL)	234.3 ± 84.5	110–520	Atrioventricular asynchronism, (n, %)
LVESV (mL)	176.9 ± 84.1	80–446	57 (78%)
LVEF (%)	27.9 ± 5.1	15–35	
LAV (mL)	98.6 ± 34.2	56–187	Intraventricular asynchronism, (n, %)
LAVi (mL/m^2^)	51.1 ± 18.6	118–22	63 (86%)
sPAP (mmHg)	41.5 ± 16	20–80	Interventricular asynchronism, (n, %)
E/A ratio	1.3 ± 0.8	0.45–4.3	58 (79%)
Valvulopaties	mild	moderate	severe
Mitral regurgitation, n (%)	11 (15%)	34 (47%)	28 (38%)
Tricuspid regurgitation, n (%)	33 (45%)	29 (40%)	11 (15%)
Aortic stenosis, n (%)	1 (1%)	1 (1%)	0
Aortic regurgitation, n (%)	12 (16%)	4 (5%)	2 (3%)

IVS = interventricular septum, LVEDD = left ventricle end diastolic diameter, LVEDV = left ventricle end diastolic volume, LVESV = left ventricle end systolic volume, LVEF = left ventricle ejection fraction, LAA = left atrium area, LAV = left atrium volume, LAVi = left atrium volume index, sPAP = systolic pulmonary artery pressure.

**Table 3:** Comparative statistical analysis between primary echocardiographical parameters at baseline and during FU.

	Before Fusion CRT-P	Follow-Up 6.4 Years ± 27 Months	*p* Value
LVEF %, mean ± SD	27.9 ± 5.1	40.4 ± 8.5	<0.0001
LVEDV (mL), mean ± SD	234.3 ± 84.5	190.1 ± 84.4	0.0019
LVESV (mL), mean ± SD	176.9 ± 84.1	123.9 ± 68.8	0.0001
LAV (mL), mean ± SD	98.6 ± 34.2	85.9 ± 27.9	0.0151
LAVi (mL/m^2^), mean ± SD	51.1 ± 18.6	45.1 ± 17.6	0.0472
sPAP (mmHg), mean ± SD	41.5 ± 16	34.1 ± 11.2	0.0015

## Data Availability

Data available on request due to restrictions (privacy and ethical reasons).
